# Change patterns of oncomelanid snail burden in areas within the Yangtze River drainage after the three gorges dam operated

**DOI:** 10.1186/s40249-019-0562-4

**Published:** 2019-06-18

**Authors:** Si-Min Dai, Jeffrey Edwards, Zhou Guan, Shan Lv, Shi-Zhu Li, Li-Juan Zhang, Jun Feng, Ning Feng, Xiao-Nong Zhou, Jing Xu

**Affiliations:** 1 National Institute of Parasitic Diseases Control and Prevention, Chinese Center for Disease Control and Prevention, 207 Ruijin Er Road, Shanghai, 200025 China; 2 Key Laboratory of Parasite and Vector Biology, Ministry of Health; WHO Collaborating Centre for Tropical Diseases; National Tropical Disease Research Center, 207 Ruijin Er Road, Shanghai, 200025 China; 3 Department of Global Health, University of Washington, Seattle, Washington USA; 4 Center for Global Public Health, Chinese Center for Disease Control and Prevention, Room 211, 155 Changbai Road, Changping District, Beijing, 102206 China; 5 Center of Disease Control and Prevention of Henan Province, 105 Nongyenan Road, Zhengzhou, 450016 Henan China

**Keywords:** Schistosomiasis, *Oncomelania hupensis*, Yangtze River, Three gorges dam, China, Operational research

## Abstract

**Background:**

An “integrated control” strategy has been implemented within seven provinces at highest risk for schistosomiasis along Yangtze River in Peoples’ Republic of China (P. R. China) since 2004. Since *Oncomelania hupensis* is the only intermediate host of the blood fluke (*Schistosoma japonicum*), controlling the distribution of snails is considered an essential and effective way to reduce the risk of schistosomiasis infection. The study aimed to determine the snail area burden and annual trend among provinces with potential risk for schistosomiasis along the Yangtze River, above and below the Three Gorges Dam (TGD).

**Methods:**

This retrospective study utilized data previously collected from the National Parasitic Diseases Control Information Management System (NPDCIMS) on annual snail surveys from 2009 to 2017. Descriptive statistics were performed for analyzing the snail burden by provinces, counties, type of environmental location and year, and mapping was conducted to present the snails distribution.

**Results:**

From 2009 to 2017, the total snail infested area decreased by 4.22%, from 372 253 hm^2^ to 356 553 hm^2^ within the seven high risk provinces. The majority of snails were found in the marshland and lake regions, outside of control embankments. The total snail burden trend remained relatively stable in upstream regions above the TGD from 2010 to 2015, while the trend decreased within downstream regions during this period. In 2016 and 2017, the total snail burden trend increased in both upstream and downstream provinces, however, upstream saw a larger increase. From 2009 to 2017, there were a total of 5990 hm^2^ of newly developed snail areas in the seven study provinces and the majority were concentrated in regions below the TGD, accounting for 5610 hm^2^ (93.70%).

**Conclusions:**

There has been a decline in total snail counts from 2009 to 2017. Meanwhile, new snail breeding areas were formed mainly within provinces downstream the TGD due to spread of snails, indicated that the oncomelanid snail would be difficult to completely eliminate. We suggest that the national schistosomiasis integrated control strategy, including mollusciding and environmental modification, will need to be enhanced significantly going forward to achieve a greater reduction in snail burden and ultimately to achieve elimination.

**Electronic supplementary material:**

The online version of this article (10.1186/s40249-019-0562-4) contains supplementary material, which is available to authorized users.

## Multilingual abstracts

Please see Additional file 1 for translations of the abstract into the five official working languages of the United Nations.

## Background

Schistosomiasis is a leading neglected tropical disease in developing countries, particularly for those living in poverty, and affects over 200 million people annually [1]. It can lead to acute gastroentestinal infections, progress to chronic hepatic, pulmonary and neurological sequale, as well as lesions in the bladder and genital area that have significant impact on health, productivity and the perpetuation of poverty [2, 3]. In the Peoples’ Republic of China (P.R. China), schistosomiasis is caused by the blood fluke, *Schistosoma japonicum*, with the snail (*Oncomelania hupensis*) as the intermediate host [4]. Because the host snail is an amphibious species and requires fresh water during its life cycle, schistosomiasis japonica is most prevalent along the Yangtze River region in China, where approximately 65 million people are at risk and at highest risk are children and workers who come in contact with water frequently. People can aquire an infection if the water was contaminated by cercarie escaped from infected snails [5].

In the 1950s, schistosomiasis was recognized as the “God of plague” and caused a substantial burden on social and economic development [6]. *O. hupensis* is the only intermediate host of schistosomiasis japonica [7]. Controlling the diffusion of oncomelanid snail is considered an essential and effective way to prevent an outbreak of schistosomiasis. The Chinese national schistosomiasis control program has taken various measures to try and reduce the snail burden within high risk provinces of China [6, 8]. A strategy called “integrated control” has been adopted by the national programme and implemented locally in high risk regions, which include: 1) improving sanitation, 2) reinforcing agricultural and hydrological development and management, 3) implementing drug treatment for infected individuals and livestocks, and 4) mollusciding, along the Yangtze River corridor [8]. In 2015, transmission control was achieved in China and by 2016 only 54 000 people were estimated to have disease, compared to 11 million in 1950s [9]. By 2017, no schistosome-infected snails were found by dissection method in China [10].

In an attempt to monitor the burden of oncomelanid snails in the Yangtze River region, there have been ongoing annual surveys. Recent data has indicated that the snails might be emerging in settings where they have not been before [11, 12]. Snail control efforts still face multiple challenges. First, with the occurrence of frequent flooding, oncomelanid snails can enter into farmland or adjacent residential zones when water levels and soil moisture are fluctuating [13]. Second, a series of large-scale water resource development projects, such as the construction of the Three Gorges Dam (TGD), the South-to-North Water Diversion Project and the Yangtze-to-Chaohu Water Diversion Project, all cross snail breeding regions, are considered as a potential risk factor for snail control [14, 15]. Finally, climate changes and ecological transformations can influence the snail breeding environment, which may cause substantial challenges affecting the elimination of schistosomiasis [16]. Several reports have suggested that the rebound of snail burden always occured earlier than an increased incidence of schistosomiasis, implying that the investigation and monitoring of the snail situation could be an effective way to predict and control transmission of schistosomiasis japonica [17, 18].

Our study proposed to determine the snail area burden among provinces with potential risk for schistosomiasis along the Yangtze River from 2009 to 2017. We explored the trend over time and the snail area burden among provinces and counties within the Yangtze River drainage, above/below the Three Gorges Dam.

## Methods

### Setting

Currently, schistosomiasis endemic areas are mainly distributed within the Yangtze River basin, which consists of seven provinces at highest risk for schistosomiasis. These provinces include: Sichuan, Yunnan, Jiangsu, Hubei, Anhui, Jiangxi and Hunan. The seven provinces have achieved transmission control, but continue to struggle towards complete schistosomiasis elimination, largely because of the persistence of the host snail that resides in the waterways of the Yangtze River. Major endemic foci occur in lakes and marshlands in eastern and central China. In general, embankments are constructed artificially in the lakes and marshlands regions for flood control. Inner embankments are the areas where residents live and work while the embankments outside refer to lakes and marshlands [19]. Hilly and mountainous regions are the schistosomiasis endemic foci areas primarily in Sichuan and Yunnan provinces.

The TGD is located in the upper reaches of the Yangtze River, the middle and lower reaches of the river are the largest endemic area of schistosomiasis in China [20]. Of the seven high risk provinces, two (Sichuan and Yunnan) are upstream of the TGD while the remaining are downstream. In this study for comparison purposes we divided these seven provinces into two groups: upstream and downstream provinces.

### Data source & variables

Data was collected from the National Parasitic Diseases Control Information Management System (NPDCIMS) on the annual snail survey between 2009 and 2017. The NPDCIMS database has been operational since 2009. The NPDCIMS consists of schistosomiasis-linked information, including data on endemic areas, monitoring of human and livestock infections and national snail survey data (including snail area and snail density).

Monitoring data on snail area status is collected in a standardized manner by regional institutions at different levels and sent annually to NPDCIMS, which is coordinated by the National Institute of Parasitic Diseases and the Chinese Center for Disease Control and Prevention (CDC). Snail area surveys are performed mainly in spring but also in autumn as a supplementary investigation in someplace where snail survey is not completed in spring. Systematic sampling method and/or environmental sampling method were used to conduct snail survey by using a frame with area of 0.1m^2^ [21]. Our study focused upon the following variables that are collected in the NPDCIMS national survey database, see Table 1.

**Table 1:** Selected National Parasite Control Information Management System variables, China, 2009–2017

Field survey variables	Snail area variables
Province name	Area of detected snails
County name	Area of detected snails first time
Reporting year	Total snail area
	Area of marshland/lake region inner embankment
	Area of marshland/lake region outside embankment
	Area of plains region with waterway network
	Area of hilly/mountainous region

### Snail area calculation

Calculation of areas detected with snails varied according to the environmental geography. A previously validated formula was used where area (m^2^) = length (meters) × width (meters) to determin snail burden [22]. The length and width were determined based on the largest distance between two frames which found live snails in the surveyed enviroments. For hilly and mountainous regions, the length was extended 15 m from each end of the frame, while width was measured according to the distance from the shoreline to water level. For lakes and marshlands, length was extended 50 m from the end of the frame with live snails detected in it. For marshlands within size of 15 ha (hm^2^), the entire area was regarded as one snail unit if snails were observed.

### Statistical analysis

Data was abstracted using a previously validated search algorithm and entered into Microsoft Excel 2017 (Redmond, Washington, USA). The data was then analyzed using SAS, version 9.4 (Statistical Analysis System, Cary, North Carolina, USA). Descriptive statistics with proportions and mapping were performed for distribution of the snail area burden by provinces, counties, type of environmental location and year. Mapping was completed using ArcGIS software version 10.4 (Environmental Systems Research Institue, Redlands, California, USA). Difference value was calculated according to counties, provinces, and upstream and downstream of the TGD by year.

## Results

### Current snail coverage and burden

In 2017, the total area of the oncomelanid snails within seven provinces (Sichuan,Yunnan, Jiangsu, Hubei, Anhui, Jiangxi and Hunan) along the Yangtze River region reached 356 553 hm^2^. Among them, lakes and marshlands occupied 344 337 hm^2^, in which 90.60% were outside of the embankment areas and 5.97% were inside embankments, accounting for 96.57% of the total snail burden, see Table 2. The area of snails in the plains regions with waterway networks, as well as hilly and mountainous regions, were 106 hm^2^ and 12 109 hm^2^, respectively, accounting for 0.03 and 3.40% of the total snail area.

**Table 2:** Distribution of snail area burden (hm^2^) by environmental location in seven provinces in China, 2009–2017

Year	Total snail area	Marshlands and lakes				Plain regions		Hilly and mountainous regions	
		Inner embankment		Outside embankment					
	No.	No.	%	No.	%	No.	%	No.	%
2009	372 253	21 100	5.67	336 764	90.47	202	0.05	14 187	3.81
2010	373 516	21 138	5.66	337 679	90.41	195	0.05	14 504	3.88
2011	372 595	20 734	5.56	338 399	90.82	176	0.05	13 286	3.57
2012	368 633	20 382	5.53	335 507	91.01	209	0.06	12 535	3.40
2013	365 366	20 090	5.50	332 537	91.01	203	0.06	12 536	3.45
2014	364 245	22 345	6.13	329 658	90.50	145	0.04	12 096	3.32
2015	356 212	20 799	5.84	323 277	90.75	111	0.03	12 025	3.38
2016	356 752	21 291	5.97	317 884	89.11	133	0.04	17 444	4.89
2017	356 553	21 285	5.97	323 053	90.60	106	0.03	12 109	3.40

Within the areas upstream of the TGD there were two provinces with 2800 hm^2^ (0.79%) of snail areas reported, characterized as hills and mountains, while provinces downstream had 353 752 hm^2^ (99.21%) reporting snail areas, with 99.21% of areas categorized as lakes and marshlands.

From 2009 to 2017, the top ten counties for snail burden were Yuanjiang, Hanshou, Yuyang, Xiangyin, Poyang, Junshan, Nanchang, Yugan, Huarong and Duchang, which lie primarily within Hunan and Jiangxi provinces.

### Trend and change of snail burden during 2009–2017

The size of the total snail area within the seven study provinces decreased from 372 252 hm^2^ in 2009 to 356 212 hm^2^ in 2015, a decline of 4.21%. In 2016 and 2017, there was an increase in snail burden, compared with 2015, ranging to 356 752 hm^2^ and 356553hm^2^ respectively. Although these two years of snail area burden were lower than in 2009, it presented an increase compared with data reported in 2015, see Table 2. By 2017, the total snail area was mainly distributed in Hunan Province (173 130 hm^2^, 48.56%), followed by Hubei Province (68 282 hm^2^, 19.15%). Within nine years, 96.28% of the total snail areas were within the lakes and marshland regions, the majority being outside the embankment areas (90.52%).

**Fig. 1:**
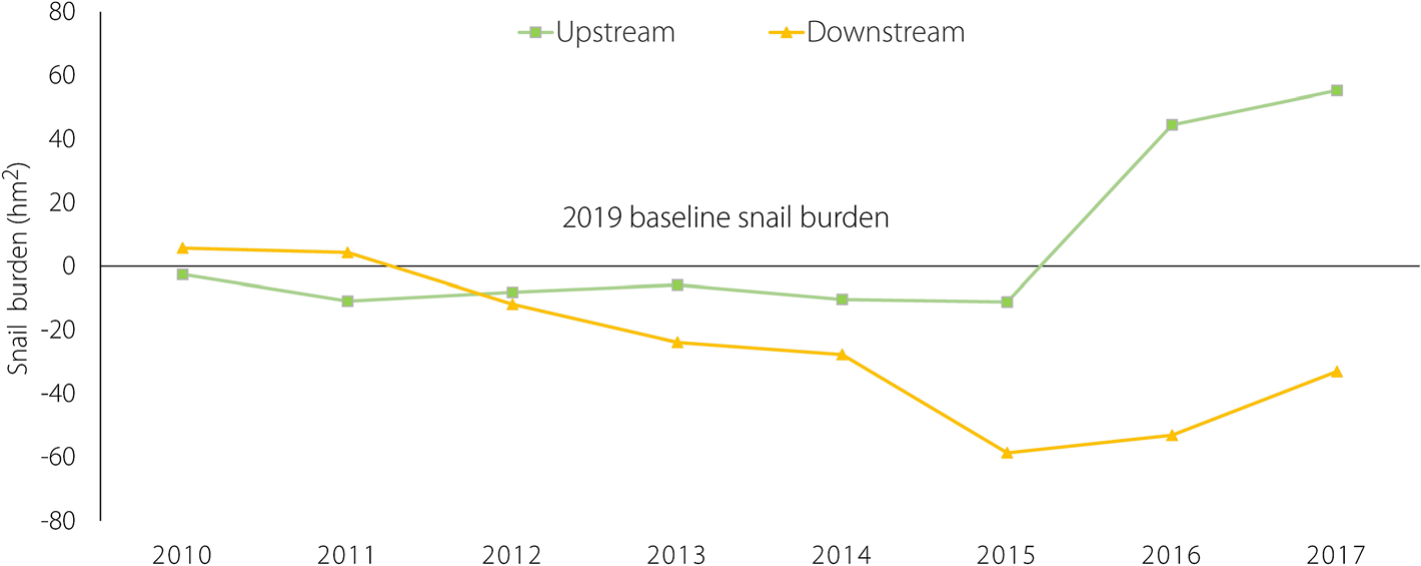
Comparison of snail burden (hm^2^) from 2009* versus 2010–2017, by province above and below the Three Gorges Dam, China. *The snail burden counts from 2009 were utilized as a baseline (0) in comparison to the following years. Upstream = above the Three Gorges Dam, downstream = below the dam

Figure 1 shows the change of the total snail area by province above and below the TGD by year. Compared with 2009, from 2010 to 2015, the total snail area of two provinces in the upstream region were relatively stable, at approximately 1500 hm^2^ and 2500 hm^2^ respectively. However, the size increased significantly in 2016 and 2017, exceeding the total snail area of 2009. Compared with those regions upstream of the TGD, the downstream regions had a greater change in total snail area. The change trend of total snail area of the lower five provinces was consistent with the trend of the entire country, indicating a gradual decrease from 2010 to 2015 and an obvious increase in 2016 and 2017.

### Trends of newly infested snail areas during 2009–2017

From 2009 through 2017, there were a total of 5990 hm^2^ of newly developed snail areas found in 63 counties in the seven study provinces, with 3638 hm^2^ in Anhui Province, 1373 hm^2^ in Hunan Province and 505 hm^2^ in Hubei Province. All of these areas were dominated by lakes and marshlands. Over the nine-year period, the newly developed snail areas were primarily distributed below the TGD, accounting for 5610 hm^2^ (93.70%), while two provinces above the dam had only 380 hm^2^ (6.30%) areas detected snails first time.

**Fig. 2:**
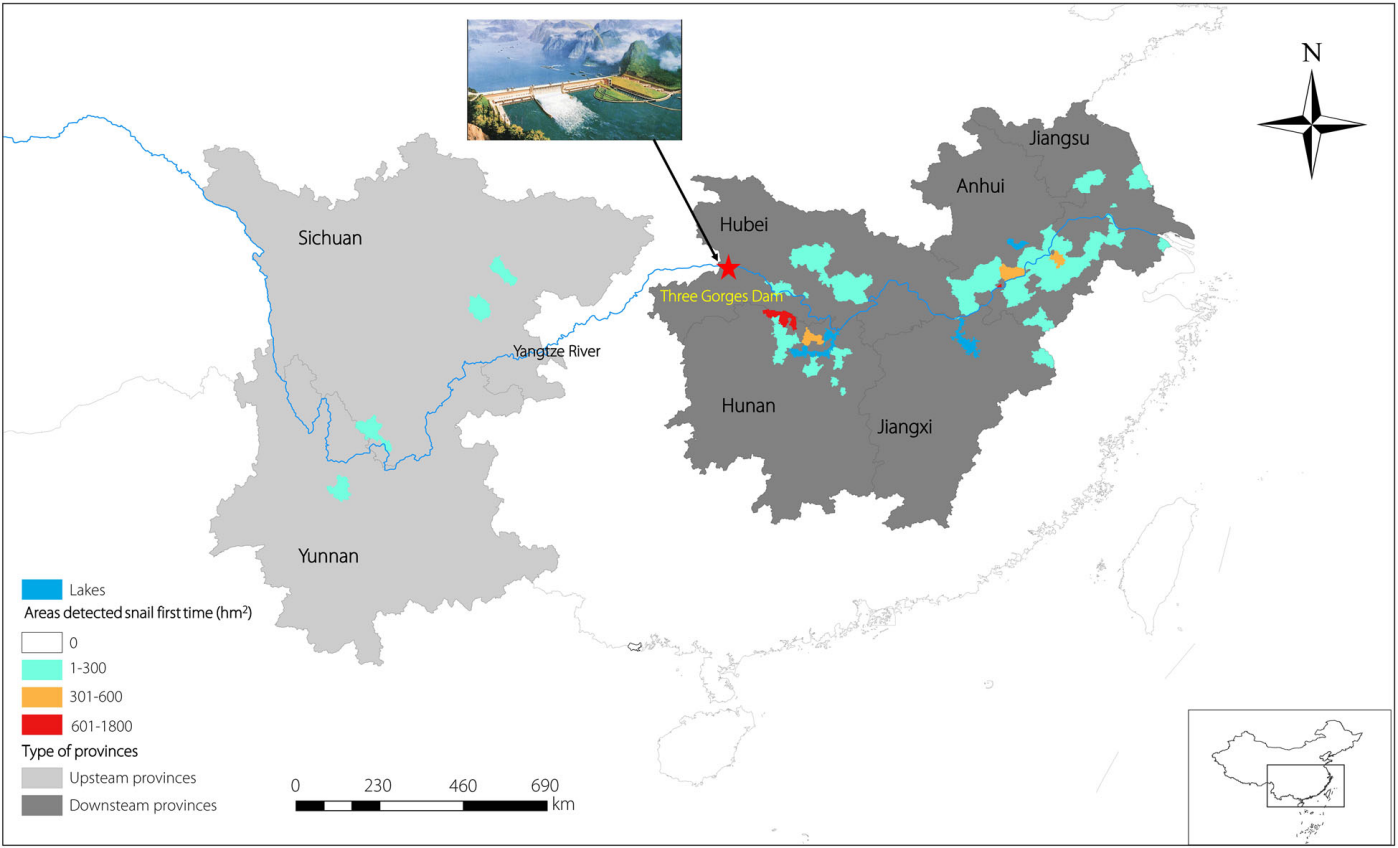
Distribution of areas detected with *Oncomelania hupensis* first time among seven provinces under schistosomiasis transmission control in China, 2009–2017

From 2009 to 2017, the area of newly found snails fluctuated, with 2016 (1345 hm^2^) having the largest increase. Figure 2 shows the distribution of the areas where snails were detected for the first time among the seven provinces. The area of detected snails for the first time was concentrated in the Hunan and Anhui provinces, which consisted mainly of lakes and marshlands, with a total of 60 counties. The accumulated area in these two provinces was 5011 hm^2^, accounting for 93.65% of the total newly found snail areas in nine years. Compared with downstream, only three counties in the upstream regions had newly found snail areas, accounting for 6.35% of the total within the seven provinces, see Fig. 2. In terms of area detecting snails for the first time, the top ten counties were Daguan, Li, Wuhu, Zongyang, Pingnan, Xiangyun, He, Xuanzhou, Miluo and Susong. Among them, only Xiangyun County, belonging to the Yunnan Province, was located upstream of the TGD.

## Discussion

Our study had the following key findings: 1) during 2009–2017 the total oncomelanid snail infested area decreased by 4.22% within the seven high risk provinces in the Yangtze River drainage and the majority of snail areas resided in the marshlands and lakes regions outside of control embankments; 2) the total snail burden trend remained relatively stable in the upstream regions from 2010 to 2015 while the trend decreased within downstream regions during this period; 3) newly found snail areas were mainly concentrated in the downstream regions.

According to our results, the total oncomelanid snail area within seven endemic provinces has declined from 2009 to 2017. However, from 2016 to 2017, the area of the oncomelanid snails in both the upstream and downstream regions increased. There are two likely reasons for this increase. Firstly, a nationwide snail survey was conducted in 2016, accelerating and strengthening snail inspection within the study provinces. This may have led to increased reporting within the national data system. Secondly, in 2016 the country experienced heavy rainfall with the middle and lower reaches of the Yangtze River suffering significant flooding, while in 2017 flooding happened in the hilly and mountainous regions above the TGD [23]. The snail habitat likely increased during the flooding and possibly led to increased reported new areas of snail burden.

Previous studies have revealed that flooding-induced high water levels can lead to expanded snail habitat and secondary schistosomiasis outbreaks [24]. It has been previously reported that the annual average snail area and the newly found snail areas during flooding years, were 2.2–2.6 times higher than normal years [25]. Because flooding may affect the distribution of the snails for the next 3–5 years, the total area of the oncomelanid snail will likely increase over the next several years. Further surveillance should be strengthened to monitor the trend of distribution and infection incidence.

Our study found that from 2009 to 2017, the snail areas were predominantly outside embankments in lakes and marshlands regions, accounting for approximately 90.06% of the total snail burden. These findings suggest that the integrated interventions were effective against snail habitats and snails have been largely kept away from the closer proximity of residents, which likely reduces the infection risk for both people and livestock.

In this study, the Three Gorges Dam acted as a unique geographical boundary. The dam was established in the largest endemic area of schistosomiasis within China. The dam’s potential impact on the transmission of schistosomiasis within the Yangtze River regions has invoked concerns previously [26]. Some studies have shown that the “winter water and summer land” environment formed by the dam is opposite to the “winter land and summer water”, and is not favorable for the survival and breeding of snails [14].

Other studies have reported that the regulation and operation of the TGD influences the water level of lakes (including the Dongting and Poyang lakes) in the middle and lower reaches of the Yangtze river by regulating water flow. Intensive debate is still going on the impact of the TGD on the distribution of snails [20, 27]. Our results suggest the operation of the TGD, combined with the ongoing measures for snail control, have stabilized the distribution of snails within endemic area. Other National water conservancy projects, including the South-to-North Water Diversion Project and the Yangtze-to-Chaohu Water Diversion Project, have been discussed widely regarding their impact on the distribution of *O. hupensis*. Some studies suggested that the multi-stage pumping technique used in these projects will not cause increased snail distribution while others disagree [15, 28, 29]. Our study provides a distribution trend of the oncomelanid snails from 2009 to 2017 that may assist other research on the effects of water transfer projects. Anyway, surveillance should be strengthened to assess the impact of large water conservancy projects on snails distribution.

From 2009 to 2017, newly found snail infested areas in the study provinces were found in 63 counties and the distribution showed certain characteristics. First, downstream from the TGD, the total area of newly developed snail accounted for 93.65% of all areas. Snails were predominantly found in the lake and marshland areas. In contrast, the upstream regions are primarily hilly/mountainous and less sustainable for new snail habitat. At the same time, snail aggregation also appeared in a province. For example, in 2015, Li County had newly found snail areas totalling 645 hm^2^, accounting for 100% of new snail burden in Hunan Province.

Second, among 63 counties with newly discovered snail areas in seven provinces, many of them changed from historic non-endemic areas to new schistosomiasis endemic environments. According to reports collected from local schistosomiasis controlling entities, due to various natural or man-made factors, the intermediate host can be imported from endemic areas through water or other transport vectors [30]. For instance, the area of newly developed snails upstream of the TGD was primarily concentrated in Xiangyun county, Yunnan province. The suspected cause for snail importation was that the host was brought in with soil, which was carried from outside regions for construction of a local expressway in 2013.

Thirdly, in many endemic counties, new snail areas were detected intermittently from 2009 to 2017. The intermittent occurence of newly found snail areas indicates that the complexity of snials control and surveillance. Many of these new snail breeding regions were close to lakes which are connecting with snails habitats. Combined with livestock herding, similar places will be at significant risk for further spread of the oncomelanid snail.

According to the latest reports from 2017, a total of 78 758 hm^2^ area was subject to snail control by using molluscicides and 5003 hm^2^ of snail habitat were treated with environmental modificationsn [10]. Nevertheless, the snail area remained elevated at 363069 hm^2^ and new snail areas were found. This further indicates that as a species, *Oncomelania* will be difficult to completely eliminate. Thus, it is not feasible to control schistosomiasis by only focusing on eliminating the *Oncomelania* host.

We need to continue to implement a comprehensive prevention and control strategy with both mollusciding and environmental modifications to condense the snails infested area. Intervetions includings replacing water buffalos with motorized tractors for farming, renovating public toilets, strengthening the inter-sectoral collaboration between governmental departments from agriculture, health, water resource development, forestry, and land resources are also encourgaged [31]. Apart from these, since the snail is impossible to be eliminated completely, monitoring the snails distribution, tendency of snails infection rate should be strengthed to provide guidance for intervention and verification of schistosomiasis elimination. At present in China, monitoring infected snail is underwent mainly by microscopy dissection method, which is not only time-consuming, but also not sufficiently sensitive [32]. A molecular diagnosis, namely loop-mediated isothermal amplification (LAMP) has been gradually used in tropical diseases, including schitosomiasis in China [33–35]. Several studies have proved the higher sensitivity of LAMP in the detection of *Schistosoma* DNA in infected snails, as well as its preferable application under field condition [32, 36, 37]. Therefore, promoting the use of such molecular diagnosis for infected snails can also help to further reduce the risk of schisotosomiasis.

To promote schistosomiasis elimination going forward, we recommend the following strategies to be considered: 1) continue oncomelanid snail surveillance within the seven endemic provinces along the Yangtze River; 2) strengthen snail habitat monitoring and reporting; 3) enhance support and further research in improving the control of the oncomelanid snail areas in different endemic envrionments, especially the lake and marshland regions.

The strength of this study is having more recent longitudinal snail data from the Yangtze River high risk regions than any previous reports that we are aware of. The primary weakness is that we have not included any comparison data regarding the incidence of schistosomiasis infections from within these regions during the study period. Additionally, there may be other environmental or human factors that we are unaware of, that could confound our results.

## Conclusions

*Oncomelania hupensis*, which is the host for a blood fluke that causes schistosomias, remains firmly established within seven high risk provinces surrounding the Yangtze River basin. The seven endemic provinces have been given high priority for snail reduction in the national schistosomiasis control strategy. However, the major habitats of the oncomelanid snail are found in lakes and marshlands, which have complex terrain, landform and water conditions. We suggest that control and monitoring strategy should be further reinforced to achieve a greater reduction in snail burden and ultimately accelerate the elimination of schistosomiasis in P. R. China.

## Additional file

Additional file 1: Multilingual abstracts in the five official working languages of the United Nations. (PDF 553 kb)

## Data Availability

The supporting data in this paper are available from the corresponding author on reasonable request.

## Abbreviations

CDC: Chinese center for disease control and prevention
NPDCIMS: National parasitic disease control information management system
P.R. China: Peoples’ Republic of China
TGD: Three Gorges Dam
