# Supplementary material for: Change patterns of oncomelanid snail burden in areas within the Yangtze River drainage after the three gorges dam operated
Source: Infect Dis Poverty. 2019 Jun 18;8:48. doi: 10.1186/s40249-019-0562-4 (PMC6580481; doi:10.1186/s40249-019-0562-4)
Supplement: Supplementary file 1 — Multilingual abstracts in the five official working languages of the United Nations. (PDF 553 kb) [file 40249_2019_562_MOESM1_ESM.pdf]

Translation of the abstract into the five official working languages of the United Nations

2009-2017؟ الصين، في اليانغ تسى نهر تصريف في مقاطعات سبع في أونكوميلاند الحلزون عبء هو ما

Si-Min Dai, Jeffrey Edwards, Zhou Guan, Shan Lv, Shi-Zhu Li, Li-Juan Zhang, Jun Feng, Ning Feng, Xiao-Nong Zhou, Jing Xu

:الخلفيه
طول على للبلهارسيات بالنسبة المخاطر لاعلي معرضه مقاطعات سبع في "المتكاملة المراقبة" استراتيجية نفذت وقد الوسيط المضيف هو هونسيس Oncomelania منذ .2004 منذ (p. r. China) الشعبية الصين جمهوريه في اليانغ تسي نهر .بالبلهارسيا الاصابه خطر من للحدوفعاله أساسيه وسيله يعتبر القواقع توزيع على والسيطرة ، الدم لحظ الوحيد البلهارسيا لخطر تتعرض ان يحتمل التي المقاطعات بين السنوي والاتجاه الحلزون منطقه عبء تحديد إلى الدراسة وتهدف (TGD). الثلاثة الخوانق سدود تحت فوق ، اليانغ تسي نهر طول على
على للسيطرة الوطنية المعلومات أداره نظام من سابقا جمعها تم التي البيانات استخدمت رجعي باثر الدراسة هذه :أساليب عبء لتحليل وصفيه إحصاءات وأجريت .2009-2017 من السنوية الحلزون الممسوحات على (NPDCIMS) الطفيلية الامراض .القواقع توزيع لعرض خرائط رسم وجري ، والسنه البيئي الموقع ونوع ، والمقاطعات ، المقاطعات بحسب الحلزون
356 553 hm^2 إلى 372 253 hm^2 من ، 4.22% بنسبة القواقع الموبوءة المساحة إجمالي انخفض ، 2009-2017 من :النتائج سدود خارج ، والبحيرات المستنقعات مناطق في القواقع غالبية على العثور تم .المخاطر عالية السبع المقاطعات داخل -2010 عام من النهر سطح فوق تقع التي المناطق في نسبيا مستقرا الحلزونية للأعباء الإجمالي الاتجاه وظل .السيطرة للأعباء الإجمالي الاتجاه زاد ، 2017وال 2016ال وفي .الفترة هذه خلال المصب مناطق داخل الاتجاه انخفض حين في ،2015 مجموعه ما هناك كان ، 2017 إلى 2009 من .أكبر زيادة شهد المنبع ان الا ، سواء حد على والمصب المنبع مقاطعات في الحلزونية عن تقل التي المناطق في الغالبية وتتركز السبعة الدراسة محافظات في حديثا المطورة حلزونال المناطق من 5990 hm^2 (93.70 ٪). 5610 hm^2 تمثل والتي ، TGD
الجديدة المناطق تشكيل تم ، نفسه الوقت وفي .2009-2017 من الحلزون التهم مجموع في انخفاض هناك كان :الاستنتاجات oncomelanid الحلزون ان إلى وأشار ، القواقع انتشار بسبب TGD المصب المقاطعات داخل رئيسي بشكل الحلزون تكاثر ذلك في بما ، المتكاملة البلهارسيات لمكافحه الوطنية الاستراتيجية ان نقترح .تماما القضاء الصعب من سيكون الحلزون عبء في أكبر تخفيض لتحقيق قدما للمضي كبير تعزيز إلى تحتاج ، البيئية والتعديلات الرخويات .القضاء لتحقيق المطاف نهاية وفي

Translated from English version into Arabic by Hamza Shaffison, through

中山大學医学院
SUN YAT-SEN UNIVERSITY SCHOOL OF MEDICINE

三峡大坝运行后长江流域钉螺分布的变化规律

戴思敏，Jeffrey Edwards，关周，吕山，李石柱，张利娟，丰俊，冯宁，周晓农，许静

摘要
引言： 自 2004 年，中国长江沿岸血吸虫病重点防治七省实施了血吸虫病综合防治措施。由于钉螺是日本血吸虫的惟一中间宿主，控制钉螺的分布被认为是降低血吸虫病传播风险的基本且有效途径之一。本研究旨在探究长江流域三峡大坝上、下游血吸虫病 7 个流行省的钉螺分布情况和年度变化趋势。
方法： 本研究为回顾性调查，数据来自 2009–2017 年国家传染病报告信息管理系统。采用描述性统计方法，从省、县、环境类型、年份等方面分析钉螺分布情况，并绘制了钉螺分布地图。

结果：2009–2017 年间，全国血吸虫病重点防治七省的钉螺总面积由 372 253 hm^2 下降到 356 553 hm^2，下降了 4.22%。钉螺主要分布在垸外的沼泽和湖泊地区。2010–2015 年三峡大坝上游两省钉螺面积相对稳定，下游五省有所减少。2016、2017 年，上游和下游省份的钉螺总面积均呈逐年增加趋势，以上游增长幅度更大。2009–2017 年，七省新发有螺面积累计达 5990 hm^2，主要集中在三峡大坝下游，共计 5610 hm^2 (93.70%)。
结论：2009–2017 年，全国钉螺总面积呈减少趋势；同时，由于钉螺扩散形成的新发钉螺面积主要分布在三峡大坝的下游五省，提示钉螺作为一个物种是很难被彻底消灭。建议今后需要大力加强包括药物灭螺和环境改造在内的血吸虫病综合防治措施，以实现更大幅度地减少钉螺负担并最终实现全国血吸虫病消除的目标。

Translated from English version into Chinese by Si-Min Dai

Densité des gastéropodes du genre Oncomelania dans sept provinces du bassin du fleuve Yang-Tsé en Chine, 2009-2017

Si-Min Dai, Jeffrey Edwards, Zhou Guan, Shan Lv, Shi-Zhu Li, Li-Juan Zhang, Jun Feng, Ning Feng, Xiao-Nong Zhou, Jing Xu

Résumé

Contexte: une stratégie de «lutte intégrée» a été déployée depuis 2004 dans les sept provinces les plus exposées au risque de schistosomiase sur le cours du fleuve Yang-Tsé, en République Populaire de Chine. Puisque *Oncomelania hupensis* est le seul hôte intermédiaire du schistosome, le contrôle de sa distribution est un moyen essentiel et efficace de réduire le risque d'infestation par ce parasite. La présente étude avait pour but de déterminer la densité de ces gastéropodes et sa tendance annuelle dans les provinces à risque potentiel de schistosomiase le long du cours du fleuve Yang-Tsé, en amont et en aval du barrage des Trois Gorges.
Méthodes: cette étude rétrospective a utilisé les données recueillies dans le Système national de gestion des informations sur la lutte contre les maladies parasitaires (NPDCIMS) lors de recensements annuels des escargots entre 2009 et 2017. Des statistiques descriptives ont été élaborées pour analyser la densité d'escargots par province, circonscription, type de biotope et année et des cartes de la distribution des escargots ont été élaborées.
Résultats: entre 2009 et 2017, la superficie totale infestée d'escargots a diminué de 4,22 %, de 372 253 hm^2 à 356 553 hm^2, dans les sept provinces à haut risque. La majorité des escargots ont été rencontrés dans les régions marécageuses et lacustres, en dehors des berges contrôlées. La densité totale des gastéropodes est restée relativement stable en amont du barrage des Trois Gorges entre 2010 et 2015 et a eu tendance à diminuer, sur la même période, dans les régions de l'aval. En 2016 et 2017, elle a augmenté dans les provinces en amont aussi bien qu'en aval, mais davantage en amont. De 2009 à 2017, leur aire de distribution a gagné au total 5990 hm^2 dans les sept provinces de l'étude, en majorité (5610 hm^2) dans les régions situées en aval du barrage des Trois Gorges (93,70 %).
Conclusions: le nombre total d'escargots du genre Oncomelania recensés a diminué entre 2009 et 2017. De nouvelles zones de reproduction se sont créées pendant cette période, notamment dans les provinces situées en aval du barrage des Trois Gorges, à cause de la propagation des escargots. Cela suggère qu'il sera difficile d'éliminer complètement *O. hupensis*. Nous suggérons que la stratégie nationale intégrée de lutte contre la schistosomiase, qui comprend des épandages de molluscicides et des modifications de l'environnement, devra être significativement renforcée pour obtenir une plus forte réduction des populations d'Oncomelania et parvenir finalement à leur élimination.

Translated from English version into French by Suzanne Assenat, Revised by Eric Ragu, through

Какова паразитарная нагрузка, вызванная моллюском рода Oncomelania, в семи провинциях в зоне осушения реки Янцзы в Китае в 2009-2017 гг.?

Si-Min Dai, Jeffrey Edwards, Zhou Guan, Shan Lv, Shi-Zhu Li, Li-Juan Zhang, Jun Feng, Ning Feng, Xiao-Nong Zhou, Jing Xu

Реферат

Общая информация: стратегия «интегрированного контроля» применялась с 2004 года в семи провинциях Китайской народной республики (далее по тексту - Китае), характеризующихся наивысшим риском шистосомоза и расположенных в бассейне реки Янцзы. С тех пор как *Oncomelania hupesis* считается единственным промежуточным хозяином кровяных сосальщиков (трематод), контроль распространения моллюсков считается неотъемлемым и эффективным способом снижения риска инфицирования шистосомозом. Исследование направлено на определение ежегодного тренда распространения области обитания моллюсков в провинциях с наивысшим риском шистосомоза, расположенных в бассейне реки Янцзы, выше и ниже гидроэлектростанции Три ущелья (TGD).

Методология: в данном ретроспективном исследовании использованы данные, предоставленные Информационной системой управления и контроля национальных паразитарных заболеваний (NPDCIMS) в ежегодных исследованиях моллюсков за 2009-2017 годы. Описательные данные представлены для анализа паразитарной назгрузки моллюсков в разрезе провинций, округов, природных зон и лет. Дана карта для обозначения зон распространения моллюсков.

Результаты: за период 2009-2017 гг. территория обитания моллюсков сократилась на 4,22 %, с 3 722,53 км^2 до 3 565,53 км^2 в семи провинциях с наивысшим риском. Большинство моллюсков было обнаружено в регионах с болотистой местностью и озерами, не имеющих контролируемой береговой зоны или насыпи. В целом паразитарная нагрузка со стороны моллюсков оставалась относительно стабильной в течение 2010-2015 гг. в регионах, расположенных выше по течению от гидроэлектростанции Три ущелья (TGB), в то время как в регионах, расположенных ниже по течению от гидроэлектростанции, распространенность моллюсков снижалась в течение данного периода. В 2016 и 2017 годах совокупная распространенность моллюсков имела тенденцию роста как в провинциях, расположенных выше по течению, так и в расположенных ниже по течению. Однако, в областях, расположенных выше по течению, рост был более значимым. С 2009 по 2017 годы было выявлено 5990 км^2 новых зон обитания моллюска в семи изучаемых провинциях и большая часть из них, насчитывающая 5610 км^2 (93,7 %), была сосредоточена в областях, расположенных ниже по течению от гидроэлектростанции Три ущелья (TGB).

Вывод: в целом произошло снижение популяции моллюсков за период 2009-2017 гг. В то же время, новые ареалы размножения моллюсков были сформированы в провинциях, расположенных ниже по течению от TGD, в соответствии с распространенностью моллюсков, что показывает вероятную сложность полного уничтожения моллюсков рода Oncomelania. Мы полагаем, что применение национальной интегрированной стратегии контроля шистосомоза, включающей применение химических средств борьбы с моллюском и модификацию биотопа его обитания, должно быть значительно расширено для достижения большего снижения паразитарной нагрузки со стороны моллюска и, в конечном итоге, его полного уничтожения.

Translated from English version into Russian by Marina Zhukova, proofread by Michael Orlov, through

¿Cuál es la carga de caracoles Oncomelania en siete provincias de la cuenca de drenaje del río Yangtzi en China, 2009-2017?

Si-Min Dai, Jeffrey Edwards, Zhou Guan, Shan Lv, Shi-Zhu Li, Li-Juan Zhang, Jun Feng, Ning Feng, Xiao-Nong Zhou, Jing Xu

Resumen

Antecedentes: desde 2004 se ha aplicado una estrategia de «control integrado» en siete provincias que están en mayor riesgo de esquistosomiasis a lo largo del río Yangtzi en la República Popular China (R. P. China). Dado que la especie *Oncomelania hupensis* es el único huésped intermediario de las duelas sanguíneas, el control de la distribución de caracoles se considera una forma esencial y eficaz para reducir el riesgo de infección por esquistosomiasis. El objetivo del estudio fue determinar la carga del área de caracoles y la tendencia anual en las provincias con riesgo potencial de esquistosomiasis a lo largo del río Yangtzi, arriba y abajo de la presa de las Tres Gargantas (PTG).

Métodos: el presente estudio retrospectivo utilizó datos previamente recopilados del Sistema Nacional de Gestión de Información sobre el Control de Enfermedades Parasitarias (NPDCIMS, por sus siglas en inglés) sobre los estudios anuales de caracoles entre 2009 y 2017. Se realizaron estadísticas descriptivas para analizar la carga de caracoles por provincias, países, tipo de ubicación en el ambiente y año; asimismo, se elaboraron mapas para presentar la distribución de los caracoles.

Resultados: entre 2009 y 2017, el área total infestada de caracoles disminuyó en un 4,22 %, de 372 253 hm^2 a 356 553 hm^2 en las siete provincias de alto riesgo. La mayoría de los caracoles se encontraba en las regiones de pantanos y lagos, fuera de los terraplenes de control. La tendencia de la carga total de caracoles se mantuvo relativamente estable en las regiones río arriba de la PTG entre 2010 y 2015, mientras que la tendencia disminuyó en las regiones río abajo durante el mismo periodo. En 2016 y 2017, la tendencia de la carga total de caracoles aumentó tanto en las provincias río arriba como en las río abajo; sin embargo, las primeras tuvieron un aumento mayor. Entre 2009 y 2017, se desarrolló un total de 5990 hm^2 de áreas nuevas de caracoles en las siete provincias del estudio y la mayoría estuvo concentrada en las regiones abajo de la PTG, lo que representa 5610 hm^2 (93,70 %).

Conclusiones: entre 2009 y 2017 se produjo una disminución en los recuentos totales de caracoles. Mientras tanto, se formaron nuevas áreas de reproducción de caracoles, principalmente en las provincias río abajo de la PTG debido a la propagación de los caracoles, lo que indica que será difícil eliminar por completo el caracol *Oncomelania*. Recomendamos que la estrategia nacional de control integrado de la esquistosomiasis, incluidos los molusquicidas y la modificación medioambiental, se mejore de manera significativa en el futuro a fin de conseguir una mayor reducción de la carga de caracoles y, en última instancia, lograr la eliminación.

Translated from English version into Spanish by Mayra León, proofread by Lindsey Hoemann, through
